# A Personalized Approach to Determining the Caloric Needs of Children with Prader–Willi Syndrome Treated with Growth Hormone

**DOI:** 10.3390/jcm12123967

**Published:** 2023-06-10

**Authors:** Yolanda Couto-Rosende, Diana Garcia-Tirado, Mónica Palacio-Marco, Assumpta Caixàs, Raquel Corripio

**Affiliations:** 1 Paediatric Digestive Disease Department, Institut d’Investigació i Innovació Parc Taulí I3PT, Parc Taulí Hospital Universitari, Universitat Autònoma de Barcelona, Parc Taulí 1, 08208 Sabadell, Spain; 2 Paediatric Endocrinology Department, Institut d’Investigació i Innovació Parc Taulí I3PT, Parc Taulí Hospital Universitari, Universitat Autònoma de Barcelona, Parc Taulí 1, 08208 Sabadell, Spain; 3 Endocrinology and Diabetes Department, Institut d’Investigació i Innovació Parc Taulí I3PT, Parc Taulí Hospital Universitari, Universitat Autònoma de Barcelona, Parc Taulí 1, 08208 Sabadell, Spain

**Keywords:** Prader–Willi syndrome, growth hormone, caloric intake, childhood, dietary record

## Abstract

Prader–Willi Syndrome (PWS) is the most frequent cause of genetic obesity. Early reports indicate that children with PWS require 20–40% fewer calories than healthy children to maintain adequate growth. Growth hormone treatment for children with PWS, approved in 2000, affects the body composition and probably affects energy requirements. This retrospective cross-sectional study analyzed the caloric intake in children with PWS aged from 6 months to 12 years old who underwent growth hormone treatment, comparing the patients’ caloric intake calculated from parent-recorded dietary intake versus the recommended caloric intake for healthy children, taking into account the age, sex, height, weight, and physical activity. We analyzed the data from 25 patients (13 (52%) boys; mean age, 6.72 ± 2.81 y; median age at starting growth hormone treatment, 1.4 y (IQR: 0.78–2.29); 17 (68%) normal weight and 8 (32%) overweight or obese). The mean daily energy intake was 1208 ± 186 kcal/d, representing 96.83% ± 18.66 of the recommended caloric intake for healthy children. The caloric intake in children with PWS treated with growth hormone was very similar to that recommended for healthy children; thus, we should rethink the dietary recommendations for these children.

## 1. Introduction

Prader–Willi syndrome (PWS) is a complex genetic disorder that affects approximately 1 in 15,000 people. PWS results from the lack of expression of genes in the 15q11-q13 region of the paternal chromosome due to paternal deletion, maternal uniparental disomy, or imprinting defects [1].

PWS is characterized by severe hypotonia at birth, feeding difficulties, overall developmental delay, hypogonadism, and growth hormone deficiency associated with short stature. Patients with PWS also have characteristic facial features and behavioral problems, including compulsions, tantrums, and self-harming behaviors [1,2].

PWS is characterized by different nutritional phases. Before birth, PWS fetuses have decreased fetal movements and restricted growth resulting in low birth weight compared to siblings. In early infancy (0–9 months), children with PWS have hypotonia and weak sucking that leads to nutritional deficiencies and failure to thrive. In later infancy (9–24 months), improved appetites and better feeding results in normal-paced weight gain. From two years of age, children with PWS begin to show a progressively increasing interest in food and preoccupation with food acquisition and start to gain weight. Once in school (mean age, 8 years), hyperphagia, lack of satiety, and obesity appear and predominate until adulthood. Some people with PWS develop satiety and resolve their food-seeking behaviors in adulthood [3].

Obesity in people with PWS differs from ordinary obesity [4]: obese people with PWS have less lean body mass and more body fat than subjects with ordinary obesity with a similar body mass index (BMI) [5]. Moreover, the total energy expenditure is lower in people with PWS, due to their low fat-free mass or low lean body mass, low levels of physical activity, and low baseline metabolic rate [6,7]. In the absence of interventions, people with PWS develop morbid obesity, which is the most important health problem in this population and one that is related to increased morbidity and mortality [8]. Weight control is one of the cornerstones of treatment for PWS. In children with PWS, weight maintenance requires the ingestion of 8 kcal–11 kcal per cm of height per day, which is 20–40% less than the recommended caloric intake for children without PWS; furthermore, restricting ingestion to 7 kcal per cm of height per day results in weight loss in children with PWS [9,10,11].

People with PWS have low weight and body length during infancy, and most have a severely reduced pubertal growth spurt. Most people with PWS have growth hormone (GH) deficiency [12]. GH deficiency is associated with a decrease in lean mass, an increase in body fat mass, hypotonia, low energy expenditure at rest, and decreased movement and tolerance to exercise [13,14]. Yet, hypotonia is central to the syndrome and persists in adulthood.

GH treatment is effective in counteracting obesity in children with PWS by increasing lean mass and decreasing body fat mass, and improving muscle tone, exercise tolerance, and energy expenditure at rest [13]. GH treatment for people with PWS was approved by the FDA in 2000 and by the EMEA in 2001, mainly to improve body composition [10,11,12,13,14]. Thereby, the use of growth hormone has changed the natural history of the disease.

Preventing obesity in people with PWS requires a combination of dietetic, physical, psychological, and behavioral interventions. It is recommended that people with PWS follow a specific program consisting of the restricted intake of calories (classically, 20–40% less than healthy children), a balanced diet, and, if necessary, strict control of access to food. Despite the importance of ensuring appropriate intake of calories, there is little evidence to support the current recommendation for restricted caloric intake in patients treated with GH, especially those aged less than 2 years. Treatment with GH has beneficial effects on height and body composition. Theoretically, the appropriate intake of calories could be different for patients with PWS undergoing GH treatment. Clinically, patients with PWS who begin GH treatment before 2 years of age show decreased BMI during the initial months of treatment.

The current study aimed to determine the appropriate intake of calories in children with PWS undergoing GH treatment in relation to the recommended intake for healthy children. It also aimed to compare the intake of calories and nutritional status between children with PWS who begin GH treatment before 2 years of age and those who begin GH treatment after 2 years of age.

## 2. Methods

### 2.1. Study Design and Population

This retrospective cross-sectional descriptive study included all children with genetically diagnosed PWS aged from 6 months to 12 years who underwent GH treatment at a single reference center for patients with PWS in Spain between June 2021 and July 2022. Patients with concomitant disease affecting dietetic intake were excluded. 

### 2.2. Variables

We recorded the following variables: daily ingestion (kcal, % carbohydrates, % fats, % protein, and grams of protein/kg body weight/d), sex, age, age at the start of GH treatment, weight, height, physical activity, and total energetic expenditure (TEE).

### 2.3. Measurements

#### 2.3.1. Auxological Variables

A single investigator measured height (patients ≥ 2 years old) or length (patients ≥ 2 years old) to the nearest 0.1 cm with a stadiometer (Harpenden, Holtain Ltd., Crosswell, UK) and body weight to the nearest 0.1 kg with a balance scale, with patients wearing light clothes without shoes. These variables were measured twice, and the mean of the two determinations was used for all analyses. Adiposity was evaluated by BMI, calculated as weight (in kg) ÷ height^2^ (in m) and BMI z-score. Patients with BMI z-scores within 1 standard deviation of the reference for age and sex were classified as normal weight, those with z-scores between 1 and 2 standard deviations as overweight, and those with z-scores > 2 standard deviations as obese [15].

#### 2.3.2. Daily Caloric Intake

As recommended [16], we analyzed the food intake for 4 days (3 weekdays and Saturday or Sunday). A pediatric nutritionist provided the patients’ families with a kitchen scale (Adler, AD-3138W) with a precision of 1 g and with instructions to immediately register all foods ingested.

To obtain patients’ daily intake of kilocalories, grams of protein and percentages of protein, carbohydrates, and fats in the food ingested, we used a nutritional calculation program (Odimet^®^, UDyTEMC). We calculated the mean number of kilocalories ingested by each patient (expressed as kcal/day).

To calculate the percentage of the recommended caloric intake (RCI) (i.e., the difference between the mean caloric intake and the total estimated energy requirement) for each patient, we used the following formula: %recommended caloric intake = (mean daily caloric intake/BMR) × 100.

#### 2.3.3. Physical Activity

Patients’ families recorded the patient’s physical activity. To calculate the activity factor, we considered the overall type of activity [17]:

Bed rest: lying in bed.

Sedentary: activity consisting of only eating, sleeping, and walking.

Light—moderate: 30–60 min per day of moderate activity in addition to sedentary activities.

Active: ≥60 min per day of moderate activity in addition to sedentary activities.

Very active: ≥60 min per day of vigorous activity in addition to ≥60 min per day of moderate activity and sedentary activities or ≥120 min of moderate activity in addition to sedentary activities (Table 1).

#### 2.3.4. Total Energy Expenditure

To calculate the baseline metabolic rate (BMR), we used the Henry equation [18], taking into account the patient’s sex, age, weight, and height. BMR was recorded as kilocalories per day. For overweight and obese children, we calculated the weight at the 50th percentile for their height.

For healthy children, total energy expenditure was estimated with the formula recommended by the European Food Safety Authority in 2013.

### 2.4. Total Energy Expenditure = Henry BMR × Activity Factor Ethics

The study fulfilled the requirements of the Helsinki declaration and the Spanish Biomedical Investigation Law 12/2007. Patients’ parents provided written informed consent, and the center’s ethics committee approved the study protocol (reference code 2022/5046).

### 2.5. Statistical Analysis

Unless otherwise indicated, categorical variables were expressed as frequencies and percentages and continuous variables as means and standard deviations. For the analyses, patients were divided into two groups according to BMI z-scores: normal weight (−2–1 standard deviation from the age- and sex-based mean) and overweight (>1 standard deviations from the age-and sex-based mean), and into two groups according to the age at the start of GH treatment (<2 years vs. ≥2 years). To compare variables between groups, we used Fisher’s exact test. Significance was set at 0.05.

All analyses were performed with SPSS Statistics for Macintosh Version 28.0 (Released 2021; IBM Corp., Armonk, NY, USA).

## 3. Results

A total of thirty patients met the inclusion criteria; five of these were excluded because their food intake records were unavailable. The excluded patients did not differ from the rest of the cohort in age, sex, or BMI. Thus, we included twenty-five patients (52% boys; mean age, 6.72 ± 2.81 years) (Table 2); of these, twenty-three (92%) were sedentary and two (8%) had light-to-moderate activity.

**Figure 1:**
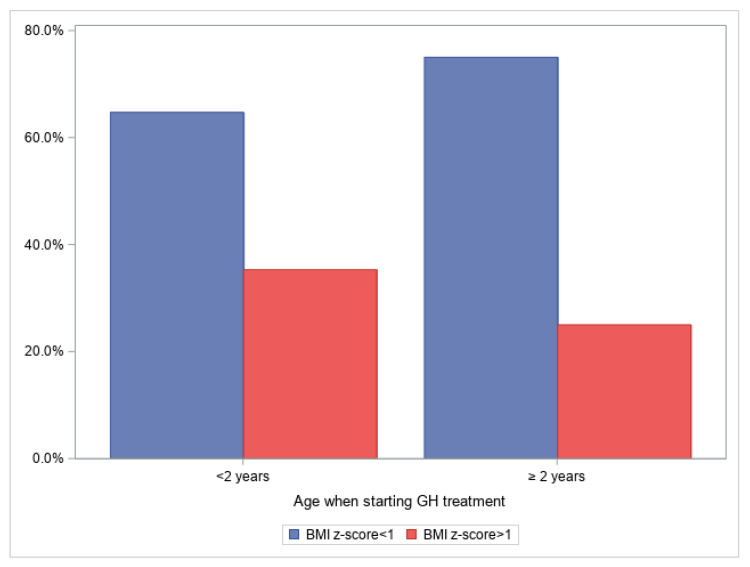
Percentage of patients with normal vs. overweight BMI z-scores according to GH treatment starting before or after 2 years old. BMI: body mass index, GH: growth hormone.

The patients’ weight was classified as normal in seventeen (68%) patients and as overweight in eight (32%). The median age at the start of GH treatment was 1.4 years (range: 5–39 m); seventeen (68%) patients were <2 years old and eight (32%) were ≥2 years old. Figure 1 shows the BMI z-score distribution according to the age at which GH treatment started (*p* = 0.6).

**Figure 2:**
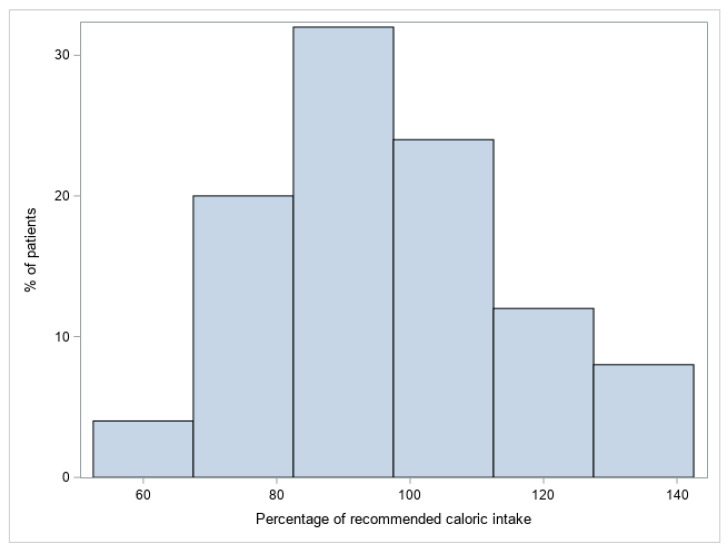
Mean percentage of recommended caloric intake for healthy children (calculated with baseline metabolic rate estimated by Henry’s equation) ingested by children with Prader–Willi syndrome, taking into account age, sex, height, weight, and physical activity.

The mean daily caloric intake was 1208 ± 186 kcal/d, and the mean recommended caloric intake was 1290 ± 317 kcal/day. The mean caloric intake by children with PWS was 96.84 (95%CI: 89.14–104.54) of the recommended intake for healthy children according to Henry’s predictive equation taking into account age, sex, height, weight, and physical activity (Figure 2).

The mean protein intake was 2.44 ± 0.92 g/kg body weight/day, representing 19.1% ± 2.90% of the total caloric intake; lipids accounted for 34.1% ± 6.55% of the caloric intake and carbohydrates for 46.8% ± 7.48%.

The mean percentage of the RCI was 98.4% ± 14.5 in the patients who started GH before 2 years of age and 93.5% ± 26.3 in those who started after 2 years of age (*p* = 0.632).

The mean percentage of the RCI was 100.7 ± 19.8% in the normal-weight group and 88.6 ± 13.6% in the overweight group (*p* = 0.132).

The mean percentage of the RCI was 102.7 ± 21.9% in boys and 90.5 ± 12.3% in girls (*p* = 0.1). Fisher’s exact test found no differences in the RCI between the normal-weight and obese groups or between the groups who started before and after 2 years of age (*p* = 1).

## 4. Discussion

To our knowledge, this was the first study to analyze the dietary intake in children with PWS initiating GH treatment early (median age, 1.4 years); in other studies, the age at initiating GH treatment ranged from 2.7 to 4 years [19,20,21]. Moreover, in the current study, the same nutritionist supervised the registry, thus, increasing the internal validity of the study.

We found that children with PWS required only 3.2% fewer calories than healthy children to maintain normal growth. These results differed substantially from those of other studies, which found that children with PWS required 20–40% less calories than healthy children to maintain appropriate growth [1,4,6]. In our study, the mean percentage of the RCI was greater than 80% in 80% of the children with PWS (Figure 1). These results agree with those of only one previous study, where the caloric intake of children with PWS treated with GH was 9% less than that of healthy children estimated with the Institute of Medicine’s equation of energy requirement [22]. Another study that, similar to ours, used Henry’s equation, found that the mean caloric intake in children with PWS was 78% (range, 41–112%) of the mean recommended intake for healthy children, although some of the subjects were not treated with GH [23]. This discrepancy suggests that differences in the mean percentage of the RCI might be influenced by the equations used to estimate the energy requirements. To minimize the impact of age, sex, weight, height, and physical activity, we used the European Food Safety Authority’s equation to estimate total energy requirements for healthy children. The low age of the children in our study might also have influenced the discrepancy in these results, since the percentage of the recommended caloric intake is lower in older children [20]. Importantly, our findings may not hold true as children with PWS age and grow, and greater caloric restriction will very likely be necessary to maintain normal weight later in life.

According to the parents’ reports of the patients’ activity levels, nearly all of our patients were sedentary. Few children were active enough to achieve a moderate physical activity score, possibly due to the young age of some patients. Moreover, we obtained these data through questionnaires; more reliable methods such as activity monitors might obtain different results. In any case, we should redouble efforts to ensure that children with PWS attain adequate activity levels.

We found no significant differences in the percentage of the recommended caloric intake ingested between the groups of normal-weight and overweight children or between the groups of children who received GH before versus after two years of age. These findings are in agreement with those reported by Garcia-Ribera et al. [24], who concluded that obesity in children and adolescents with PWS was not associated with total calorie intake. Nevertheless, our sample was very small, and we cannot know whether the overweight and/or obese patients in our study underreported their calorie intake or whether their energy requirements were lower because they have less lean body mass. Furthermore, in some cases, the parents could have restricted the patients’ caloric intake because of their weight.

The lack of significant differences in the mean percentage of the RCI between the groups of children who received GH before versus after two years of age might also be influenced by the size of our sample; a larger sample might show a larger percentage of recommended caloric intake in children who start GH treatment before two years of age. To further explore this question, it would also be interesting to do a longitudinal cohort study.

The mean percentage of the RCI also did not differ between the boys and girls. We cannot know whether this was related to the small sample size, but theoretically boys would need to ingest more calories than girls. The age of our cohort may have influenced this result because the difference in the caloric intake was lowest at a mean age of 6.72 years and increased with age.

The analysis of the distribution of proteins, carbohydrates, and fats showed that our patients followed a balanced diet within the ranges recommended for healthy children [18], although there was a trend toward ingesting less carbohydrates and more proteins than recommended. The parents of the children with PWS were generally involved in controlling their diets, and parental involvement likely helped ensure a balanced diet, as has been observed in other studies [20,24]. Miller et al. [25] found that children with PWS who followed a balanced diet achieved better weight control than those who followed a diet only limiting caloric intake, even when the total caloric intake was similar.

Many studies have been published on physical activities in children with PWS. In a systematic review on the habitual physical activities and sedentary behavior and their effects in children with PWS, Bellicha et al. [26] explored the effects of eight interventions. Overall, they found that the interventions to increase physical activity improved physical fitness, as evidenced by improved walking capacity, muscle strength, and gait, but in all but one study this failed to bring about a significant loss of weight or fat. Moreover, they found that interventions to increase physical activity seemed to have a beneficial effect on lean body mass and bone mineral density.

On the other hand, anti-obesity medications are also used to control weight in children with PWS. Goldman et al. [27] reviewed fourteen studies on drugs used in children and adolescents with PWS (three on topiramate, one on metformin, four on liraglutide, five on oxytocin, and one on naltrexone–bupropion). All the studies reported decreased hyperphagia with variable effects on the BMI without any significant adverse effects. However, regulatory agencies have approved these treatments only for older children (>10, 12, or 16 years of age), and there are no widely accepted guidelines on the acceptability, safety, or efficacy of anti-obesity medication in children with PWS [26].

Our study had some limitations. Our analyses were conducted on data from the parents’ registering the food intake, and this approach could have introduced biases. To minimize the possible biases, the parents were instructed on how to complete the registries and were considered capable of this task. Although we cannot rule out the possibility that some patients might have consumed some food without their parents’ knowledge, the food intake records are valuable tools for assessing energy intake [17]. The size of the sample in our study could have led to random errors. Because PWS is a rare disease, it is difficult to achieve large numbers of patients; however, our center is a regional reference for PWS, and including all the patients who met the inclusion criteria enabled us to achieve a sample size similar to or larger than those used in other recent studies [19,20,21,22,23]. Our study was further limited by our reliance on the BMI alone to evaluate the patients’ nutritional status. Future studies should also incorporate other anthropometric measurements such as skin folds and body perimeters. Finally, the cross-sectional design of our study precluded inferring causality.

Future studies about the energy requirements for children with PWS who start GH treatment before two years of age should include anthropometrics and indirect calorimetry tests.

## 5. Conclusions

The present study found that children with PWS ingested 96.83% (95%CI: 89.14–104.54%) of the calories that healthy children would need according to Henry’s equation, taking into account their weight, age, height, sex, and physical activity. These findings suggest that we should rethink the classical recommendation of reducing caloric intake by 20–40% in these children. An individualized approach seems to be a better way to establish the recommended caloric intake for children with PWS, especially when they are younger and under GH treatment.

## Figures and Tables

**Table 1:** Physical Activity Coefficients (PA Values) for use in equations to estimate the energy efficiency ratio (adapted from reference 17).

	Sedentary (PAL * 1.0–1.39)	Low Active (PAL * 1.4–1.59)	Active (PAL * 1.6–1.89)	Very Active (PAL * 1.9–2.5)
	Typical daily living activities (e.g., household tasks, walking to the bus)	Typical daily living activities PLUS 30–60 min of daily moderate activity (e.g., walking at 5–7 km/h)	Typical daily living activities PLUS at least 60 min of daily moderate activity	Typical daily living activities PLUS at least 60 min of daily moderate activity PLUS an additional 60 min of vigorous activity or 120 min of moderate activity
Boys 3–18 y	1.00	1.13	1.26	1.42
Girls 3–18 y	1.00	1.16	1.31	1.56
Men 19 y+	1.00	1.11	1.25	1.48
Women 19 y+	1.00	1.12	1.27	1.45

* PAL = Physical Activity Level.

**Table 2:** Subjects’ characteristics.

Variable	Statistics
Age (years)	6.72 ± 2.81
Age at start of GH (years) *	1.40 (0.78; 2.29)
Weight (kg) *	24.6 (16.6; 33.7)
Height or length (cm) *	122.0 (105.0; 137.0)
BMI (kg/m^2^) *	16.8 (15.1; 20.4)
BMI z-score *	−0.10 (−0.75; 1.50)
Caloric intake (kcal/d)	1208 ± 186
Grams protein/kg/d	2.44 ± 0.92
%Proteins	19.1 ± 2.90
%Lipids	34.1 ± 6.55
%Carbohydrates	46.8 ± 7.47
BMR (kcal/d)	1290 ± 317

Statistics represent means ± standard deviations, except those marked with an asterisk (*), which represent medians (interquartile ranges). (GH: growth hormone treatment, BMI: body mass index, BMR: baseline metabolic rate calculated with Henry’s equation).

## Data Availability

The data presented in this study are available on request from the corresponding author. The data are not publicly available.
